# Collective forward-looking responsibility of patient advocacy organizations: conceptual and ethical analysis

**DOI:** 10.1186/s12910-021-00680-w

**Published:** 2021-08-23

**Authors:** Regina Müller, Christoph Rach, Sabine Salloch

**Affiliations:** 1 Institute of Ethics and History of Medicine, University of Tübingen, Gartenstraße 47, 72074 Tübingen, Germany; 2 Department of Psychiatry, Psychotherapy and Psychosomatics, Agaplesion Markus Hospital, Wilhelm-Epstein-Straße 4, 60431 Frankfurt am Main, Germany; 3 Institute of Ethics, History and Philosophy of Medicine, Hannover Medical School, Carl-Neuberg-Str. 1, 30625 Hannover, Germany

**Keywords:** Patient groups, Collectives, Patient representation, Patient involvement, Bioethics

## Abstract

**Background:**

Patient advocacy organizations (PAOs) have an increasing influence on health policy and biomedical research, therefore, questions about the specific character of their responsibility arise: Can PAOs bear moral responsibility and, if so, to whom are they responsible, for what and on which normative basis? Although the concept of responsibility in healthcare is strongly discussed, PAOs particularly have rarely been systematically analyzed as morally responsible agents. The aim of the current paper is to analyze the character of PAOs’ responsibility to provide guidance to themselves and to other stakeholders in healthcare.

**Methods:**

Responsibility is presented as a concept with four reference points: (1) The subject, (2) the object, (3) the addressee and (4) the underlying normative standard. This four-point relationship is applied to PAOs and the dimensions of collectivity and prospectivity are analyzed in each reference point.

**Results:**

Understood as collectives, PAOs are, in principle, capable of intentionality and able to act and, thus, fulfill one prerequisite for the attribution of moral responsibility. Given their common mission to represent those affected, PAOs can be seen as responsible for patients’ representation and advocacy, primarily towards a certain group but secondarily in a broader social context. Various legal and political statements and the bioethical principles of justice, beneficence and empowerment can be used as a normative basis for attributing responsibility to PAOs.

**Conclusions:**

The understanding of responsibility as a four-point relation incorporating collective and forward-looking dimensions helps one to understand the PAOs’ roles and responsibilities better. The analysis, thus, provides a basis for the debate about PAOs’ contribution and cooperation in the healthcare sector.

## Background

Patient advocacy organizations (PAOs) have increased in their number and social visibility over the last few decades [1–3]. There are pragmatic reasons for joining forces: Individuals together have more power and better opportunities to advocate for their specific interests than alone. However, there are also moral reasons for joining a PAO, such as helping each other and campaigning for justice. Looking at the common goals and tasks of PAOs, normative values such as justice and ethical motives such as empowerment become apparent. This shows that PAOs are not only active in advocacy, but also cover ethical issues. Moreover, their activities are subject to ethical evaluations and linked with ethical concepts, such as responsibility. The involvement of PAOs in biomedical research [1, 2, 4, 5], politics [6] and industry [7, 8], for example, is seen as controversial and raises questions about the general character of their responsibility. Since PAOs are confronted with normative questions of responsibility in these exemplary fields of activity, they are expected to respond. However, it is not always clear for what, to whom and on which basis PAOs are responsible given the complex healthcare systems within which they operate.

The aim of the current paper is to analyze PAOs’ moral responsibility to provide guidance not only to themselves but also to political, scientific and industrial stakeholders. Responsibility is presented as a concept with four reference points: (1) The subject, (2) the object, (3) the addressee and (4) the underlying normative standard. This four-point relationship is applied to PAOs and the dimensions of collectivity and prospectivity are analyzed in each reference point.

## Patient advocacy organizations

### Characteristics and missions

There is a great variety of PAOs [1, 3]. They differ in size, organizational structure, level of professionalization, strategy and financial capacity. There are groups operating at the local level, while others have an international scope. Several groups are working across diseases; other groups are condition-specific [9]. Despite the diversity of the groups, many definitions describe typical attributes for PAOs, such as their nongovernmental, nonprofit and patient-driven character [1, 3, 9, 10]. The PAOs are often defined as “[…] not-for-profit organisations which are patient focused, and where patients and/or carers […] represent a majority of members in governing bodies” [11]. They usually aim at strengthening the voice of affected and sometimes overlooked individuals, and ensure that their interests are recognized [1, 3, 10]. The contribution of PAOs can, therefore, be seen as “[…] representing and voicing the situation of a specific population that would otherwise not be represented” [9]. The groups pursue this mission in various ways. Their activities cover, inter alia, interacting with patients, educational activities [9], promotion of research [2, 10] and engaging in policy and industry [7, 8]. The PAOs often bring together not only those directly affected but also related families, interested individuals, groups concerned with similar problems and professionals.

The shared mission of PAOs to advocate for those affected has its major roots in the experience of injustice, as many PAOs represent, for example, patient groups or diseases that are under-recognized, such as orphan diseases [1, 3]. Consequently, a core normative value that characterizes the work of PAOs is social justice. Moreover, the wish to help each other can be a strong motivator for affected individuals to initiate or join a PAO. Mutual support is, therefore, a further normative value strongly represented by PAOs. In addition, the normative ideal of empowerment can be found in many PAOs, for example, in statements such as ‘Strengthening the patient’s voice’ (for instance: the ‘Strengthening Patient Voices project’ by the Meningitis Research Foundation). Looking at the core values of the PAOs, the principles of justice, beneficence and empowerment (as one key aspect of autonomy) crystallize. These moral dimensions of the PAOs’ work, together with their non-profit and patient-focused character, distinguish PAOs from other organizations in healthcare, such as research institutions, professional bodies or insurances.

In contrast to profit-oriented or politically managed organizations, PAOs can be classified as civil society organizations (CSOs) due to the mentioned dimensions and characteristics. CSOs can generally be defined as non-governmental actors, varying from activists, small community-based groups and informal movements to highly organized institutions and international organizations or networks [12]. One common goal of CSOs is to participate in or influence (health) policy [13, 14] and research [15] on behalf of citizens or socially and economically disadvantaged groups, for example, women, persons with disabilities or migrants [16]. Due to their independence from direct governmental management, their non-economic aims and their voluntary and bottom-up way of working [11], PAOs and CSOs have much in common. However, as CSOs work on a wide-ranging scope of themes, from environment and trade to human rights, PAOs work in the context of healthcare and are motivated by the specific needs and values of patients.

### Challenges

The PAOs are confronted with internal and external challenges in their various fields of action and face multifaceted ethical issues. Many activities, for example, confront them with ethical questions regarding representativeness. The criteria which qualify one or more persons to represent a group are not clearly defined and PAOs typically represent various interests simultaneously, for example, of patients and families [17–19]. Additionally, PAOs need to maintain a balance between professionalization and representativeness. More intensive contact with healthcare professionals or companies is often accompanied by less time for the PAO members and eventually can result in a loss of contact with the grassroots [9]. This is accompanied by the risk that the PAOs may decide and act independent of their members and lose sight of their interests. The question of the extent to which individual patients or members can and should participate in the collective decision-making is challenging for each PAO and needs to be addressed at the level of the PAOs’ decision-making structures. The distribution of resources, tasks and responsibilities within PAOs can lead to difficult processes.

Such ethical issues arising *within* a PAO are accompanied by ethical questions occurring *between* different PAOs and other stakeholders. The involvement in politics [6] and research [4, 5] and the cooperation between PAOs and economic stakeholders [7, 8, 20] can sometimes be problematic. Building financial relationships with industrial companies, for example, can help PAOs to pursue their goals [21] but might lead to pressure to conform to the funder’s interests [20, 22]. Many organizations have committed themselves to support research. However, PAOs that want to foster biomedical research face many ethical questions, such as the extent to which they should encourage their members to participate in a study or the extent to which the specific interests of the PAO should influence the research designs [4]. Another problem for PAOs can be that external cooperation, for example, with politicians, might be characterized by tokenism [9]. Finally, given the missing access to independent and adequate resources for PAOs [9], questions regarding the fair distribution of resources arise.

These are exemplary challenges showing that PAOs are faced with various ethical questions regarding their internal structures and external activities. Focusing on these ethical issues makes the moral character of PAOs’ activities more transparent. When confronted with decisions of ethical significance, justifications of their activities and their implications are required from PAOs: Their actions are then subject to ethical evaluations and linked with the concept of moral responsibility. For example, if a PAO wants to advance biomedical research and is partnering with an economic stakeholder to achieve this goal, this PAO should be able to explicate how many funds the PAO accepts from the economic stakeholder to promote that research. By being able to answer such questions, the PAO demonstrates how it acts in a responsible manner regarding these activities.

## Moral responsibility

There are numerous definitions of moral responsibility [23–25], for example, backward- or forward-looking accounts [26] and collective [27–32] or individual approaches [33]. The concept of responsibility in healthcare and medicine has long been discussed [34], for example, different models of responsibility in bioethics [24], the individuals’ responsibility for their own health [33, 35, 36], and collective responsibility in healthcare [37–39]. The diversity of literature on responsibility makes it almost impossible even to provide a systematic overview of the main argumentative lines of the discourse. However, responsibility can be generally understood as both a causal and a normative relation [35]. Causal responsibility merely means that somebody (or something) has caused something, whereas the attribution of the consequences remains a descriptive act [23]. In the context of PAOs, the second meaning, responsibility as a normative relation, is of interest. In this meaning, “[…] responsibility refers to the demand on a person or an institution to justify its action or actions towards another person or institution” [35]. The conditions for moral responsibility, for example, free will, are controversial. However, widespread agreement exists on the following key traits: To describe an agent as responsible for an action means that this agent fulfils some epistemic conditions and conditions of control [33]. The agent must have a certain degree of awareness of the consequences of his/her action, including an understanding of their moral significance, and sufficient control over his/her action [33].

Wrongdoings are the typical occasions for asking about responsibility and the respective debates usually refer to the attribution of harm that one individual did to another individual. However, such an individualistic, negative and backward-looking understanding of responsibility does not fully meet the circumstances of PAOs’ engagement. Their activities have a collective character, do not usually focus on specific tasks but on a broad thematic issue and their orientation is prospective. Consequently, the dimensions of collectivity and prospectivity could be more appropriate for PAOs’ responsibility than the often-used conditions of individuality and retrospectivity.

### Collective dimension

Collective responsibility covers situations in which more than one individual can be seen as responsible for something. The responsibility is spread to (members of) a group instead of being bound to one individual [28]. Since many agents in the healthcare system, for example clinics or the medical professions, are groups to which the concept of individual responsibility does not fit, the concept of collective responsibility allows to make sense of collectives in healthcare without having to abandon the notion of individual responsibility. Moreover, modern medical technologies, such as human-machine cooperation, require a reflection on the collective dimension of responsibility in healthcare [40]. If healthcare systems should remain an area in which morality is a relevant factor, a way must be found to make the moral responsibility of these associations understandable. PAOs are only one of several groups that are operating in the healthcare system.

However, since the concept of collective agency and collective responsibility turns groups, as opposed to their individual members, into moral agents, it has been strongly scrutinized both methodologically and normatively in recent years [31]. Despite the comprehensive research, collective responsibility remains a contentious concept, since it is still unclear whether collectives can become (moral) agents and how collective action and intention are possible at all [27–32, 41–43].

If it is assumed that collectives can bear responsibility, the subsequent question is: how, if at all, can that responsibility be shared within the collective [28]. Some theorists argue that responsibility can only be constructed in individual terms. According to this position, the “responsibility of the group” is merely aggregated individual responsibility and the individuals in the group remain the responsible subjects [28]. The opposite opinion claims, that there is a responsibility of the group on its own and that this responsibility cannot be reduced to the individuals forming the group [28]. Peter A. French, for example, argues that collective responsibility does not entirely consist of or is exhausted by the individuals within the collective [37]. There are not only these binary counterparts, but also other models and many positions in between [39]. The current paper seizes the dispute between these two sides by examining whether a collective dimension is helpful when considering PAOs’ responsibility.

### Prospective dimension

The classical literature on responsibility usually refers to backward-looking concepts: Much of the literature focuses, for example, on responsibility as guilt [44, 45], accountability [46, 47] and liability [29, 48]. More recent accounts, on the contrary, often draw on forward-looking approaches [49, 50]. Retrospective (or backward-looking) responsibility covers something an agent has done (or omitted to do) and its consequences. It concerns activities in the past. Prospective (or forward-looking) responsibility refers to future activities, often to the occurrence (or prevention) of certain states, and means responsibility for something that is not yet the case [50]. The agent is not obliged to act in a concrete way but to behave in a way that is promoting a certain state. Forward-looking responsibility is often linked with backward-looking responsibility, but the relationship between these two types is controversially discussed [26].

The current paper focuses on the future-oriented dimension because this dimension seems more appropriate for the PAOs’ advocacy role and their caring activities. The character of PAOs’ goals are usually to change something for a better future, such as improving patient care or raising public awareness of a certain disease. The typical tasks of a PAO, such as policy, education and promoting research and development, are activities aimed at improving the conditions for the individuals affected. As PAOs usually take care of these issues voluntarily and in a patient-driven way, this article sheds light on the caring and future-oriented activities of the PAOs.

### Responsibility as a relational concept

As has been mentioned above, in the context of PAOs, the meaning of responsibility as a normative relationship is of interest. Understood as a normative relationship, responsibility manifests in relations between different reference points (relata). Due to various possible relata, the relational understanding is a useful analytical tool to analyze the complex field of PAOs’ activities. Although there are concepts using up to six [35] or seven [24] reference points, the following four relata seem—in the view of the authors—at least necessary for moral responsibility: Someone (the subject) is responsible to somebody (the addressee) for something (the object) regarding normative criteria. This four-point relationship will be applied to PAOs, each of the relata will be discussed, and the dimensions of collectivity and prospectivity in each reference point will be analyzed (Table 1).

**Table 1:** Relata of responsibility in the context of PAOs

Relata of responsibility	Context of PAOs	Dimension of collectivity	Dimension of prospectivity
Subject	PAOs	PAOs as collectivities capable of intentionality, acting and moral responsibility	Long-term structures and far-reaching goals of PAOs
Object	Patient representation and advocacy	Collective representation of a shared interest, respectively, an issue that is important for many people	Campaigning refers to future situations that are not yet the case
Addressee	From a specific (patient) group to others in the health sector and society	Direct benefits to the target group, understood as a collective, and collective, indirect benefits for others	Future patients and generations
Normative standard	Legal regulations; ethical guidelines and codices; ethical principles of justice, beneficence and empowerment	Standards that are the result of a shared deliberative process	Standards that show a certain degree of stability and long-term orientation

## Responsibility of PAOs

### The subject

The first reference point addresses the subject of responsibility and draws attention to PAOs as collectives and, therefore, to the underlying question whether collectives could be assigned moral responsibility. According to French “[…] something must, at least, be an intentional agent to be properly held morally responsible for its actions” [37]. The debates on responsibility exhibit a close systematic connection between responsibility and intentionality, but also a strong dispute about this relation [46–54]. Following French’s argumentation, some collectives are capable of intentionality and can, consequently, bear moral responsibility [37].

French differentiates between aggregate and conglomerate collectivities. A collectivity can be understood as an aggregate “[…] if the identity of that collectivity consists in the sum of the identities of the persons who comprise the membership of the collectivity” [37]. An aggregate is, for example, the people standing on the corner [37]. By contrast, “[…] conglomerates are such that their identities do not entirely consist in or are not exhausted by the identities of the persons that are associated with them” [37]. The conglomerate’s identity is insofar independent of its individual members as it is consistent with a (constantly) changing membership. An example is a clinic whose identity remains the same even if all employees change over time. The crucial factor is that conglomerates, in contrast to aggregates, have a decision procedure for determining group actions [37]. This decision structure transforms the individual intentions and acts into a corporate decision. According to French’s argument, the decision structure provides the basis for the attribution of intentionality and, consequently, moral responsibility. In line with French’s argumentation, the strategy of the current paper is to assign collective responsibility to those collectives, which have decision-making procedures, including (1) the capacities for forming intentions and (2) the capacities to act. Then, collectives qualify as moral agents and hence can be attributed moral responsibility.

Depending on their size and degree of professionalization, PAOs show the elements of French’s approach. Due to the complexities of translational activities and the integration of different subgroups, larger and internationally organized PAOs are highly structured with different levels and positions, such as boards of directors, advisory committees and administration services. In addition, most PAOs have policies, often documented in statutes or mission statements, which make clear whether a decision has been made for corporate reasons. Since PAOs have structures for determining corporate decisions, they can be understood as conglomerates and, according to French’s argument, fulfill the conditions of intentionality and moral responsibility.

In addition to the collective dimension of PAOs as subjects of moral responsibility, there is also a future-looking aspect. The prospective dimension of PAOs can be explained in terms of stability and persistence. The PAOs usually have long-term structures and pursue future-oriented goals. Moreover, when understood as conglomerates, the identity of PAOs remains even if the individual members change. Based on these long-term structures, the concept of PAOs as subjects of responsibility can be understood as extending into the future and, consequently, show the forward-looking dimension.

### The object

If PAOs are the subjects of responsibility, what are they responsible for? One way to answer this question concerns roles. Roles are often linked to specific behavior and can, therefore, help to narrow down the scope of responsibility. However, the various roles of PAOs lead to different objects of responsibility. Involvement in research, for example, is accompanied by other responsibilities than engagement in politics. However, despite the diversity of PAOs, one mission seems to be common: “Many PAOs characterize their efforts as attempts to give patients a greater voice and ensure that patients’ interests are acknowledged by those in positions of power” [10]. The PAOs typically understand themselves as advocates that represent the interests of those affected [1, 3]. This advocacy role of PAOs, although initially self-attributed, is increasingly confirmed by society and policy. The PAOs, for example, are often promoted by political organizations, such as the World Health Organization (WHO) because of their specific function to speak on behalf of patients [55, 56]. Due to this strong weighing, patients’ representation and advocacy can be seen as the primary role and, therefore, as the main object of PAOs’ responsibility. While this view does not yet provide concrete ethical obligations, it highlights the moral character of PAOs’ engagement and can encourage them to emphasize their core values—representing patients and advocating their interests. Responsibilities that are more concrete, for example, regarding certain cooperation partners can build on these basic values.

However, there are several points to consider. Firstly, due to the diversity of the tasks (e.g. policy, education, promoting research) and several interests to be represented within a PAO (e.g. patients, families, carers), it is not straightforward to specify the patient representation by a PAO in a concrete task and it is often unclear who can represent the members of the PAO adequately [17–19]. The object of PAOs’ responsibility remains to some degree unspecified because the concrete forms and implementation of patient representation are manifold, ranging from interaction with individual patients, public communication and educational activities, to political and industry engagement. Secondly, even with such a broad topic as patient representation, a limit to the scope of PAOs’ responsibility must be drawn. If issues are not covered or excluded from the domain of PAOs’ responsibility, they must be moved to the area of someone else’s responsibility in order not to be overlooked. For example, a PAO may set itself the mission of improving patient care for patients with a particular rare disease and, therefore, seek to raise awareness of that disease within medical education. However, it is not the role of the PAO to decide on the content of the medical education or to ensure the quality of the education. This remains the responsibility of the teaching institutions and the medical profession.

Finally, patient representation, for example in health politics, is the result of various activities of multiple agents and is only partially modifiable by PAOs. Consequently, PAOs should not be understood as being responsible for patient representation alone. Other stakeholders in health policy, for example, governments, political organizations such as the WHO and CSOs, whose remit can overlap with that of PAOs, should not be relieved of their responsibilities. For example, a PAO that advocates for a specific rare disease at the regional level and therefore has few members and resources might not be able to carry the overarching responsibility to represent all patients with rare diseases in international health policy. This would lie beyond the scope of that PAO and would instead be the task of international (political) bodies such as the WHO and CSOs advocating on a global level. On the national level, the PAO is also not responsible for the needs of these particular patients alone. National governments, health policy-making institutions, publicly funded healthcare systems and CSOs cannot transfer their responsibility to care for patients with rare diseases to the PAO. Regardless of these points, campaigning for a shared interest bears a collective dimension and since the relevant question “what needs to be done to help those affected?” refers to future activities and states, PAOs’ responsibility for patient representation is also prospective in its direction.

### The addressee

Having identified what PAOs are responsible for, the question of the addressee remains. Given their advocacy role, it seems acceptable that the addressee of PAOs’ responsibility is primarily their targeted (patient) group. However, only considering distinct groups of patients can be too shortsighted in some situations. Issues regarding genetic contexts, for example, might go beyond the patients and affect other individuals or groups. A PAO that supports patients with a genetically determined condition and advocates for genetic testing in childhood or pregnancy should also consider the impact of such testing on families, patient groups with other genetic conditions and society. As this example shows, PAOs are frequently confronted with issues of ethical significance that not only affect their own members but also other groups. If PAOs only take the interests of a certain patient group into account, this can lead to questionable consequences for others. It is, therefore, within the responsibility of PAOs to consider the ethical implications of their activities. This means that PAOs should be committed to a wider range of addressees, however, the question inevitably arises regarding how far the scope of the addressees should extend.

In the context of health policy, for example, Onora O’Neill emphasizes that health issues cannot be restricted to limited groups but need to be considered in a broader context [57]. She claims that measures which are targeted at certain groups can, simultaneously, have collective benefits [57]. O’Neill’s idea can be transferred to PAOs: They can be structured in such a way that they produce direct benefit for their defined target group and, in addition, indirect benefit for others. Exemplarily, although a PAO is committed to a specific disease, successfully (co-)funded basic research can help other and future patients. This does not mean that PAOs should override the interests of their target group. An expansion of the addressees, for example, to patients with similar conditions, always needs to be critically assessed. A crucial point is to find a balance between the group’s own interests and the interests of other groups. Finding this balance can be especially difficult for PAOs, as PAOs are often built bottom-up. In many cases, PAOs are driven by the individuals affected who often belong to overlooked or discriminated populations. It may be difficult for them to accept that the PAO, which was established to advocate for their specific interests, is now supposed to advocate for the interests of others. However, as argued above, health issues cannot be restricted to limited groups and it is within the responsibility of PAOs to consider the ethical implications to a broader range of potentially affected individuals. Depending on the size and structure of a PAO, the leaders or board members might be in the position to undertake the difficult task of balancing.

Other addressees of PAOs’ responsibility could be politicians, scientists and private stakeholders. Although they form a fruitful network for PAOs, such relationships, especially if they are financial, may lead to conflicts of interest and create, for example, biases in PAOs’ educational activities [7, 8, 22]. The PAOs that establish such relationships run the risk of becoming financially dependent and influenced in their activities and might fail to represent the patients’ perspective [7, 8, 21, 22]. Due to the frequent lack of independent and adequate resources for PAOs’ activities [9], PAOs are often dependent on external funding and, thus, particularly susceptible to dependencies and influences from outside. As long as patient representation is the object of a PAO’s responsibility, political, scientific and private stakeholders may be helpful network and cooperation partners for PAOs, but they do not seem to be legitimate addressees of PAOs’ responsibility because of the risk of ignoring the advocacy role and pretermitting the interests of the patients. Of course, PAOs have responsibilities towards politicians, scientists and industrial partners when they work together with them, for example, to keep agreements, but these responsibilities are not the subject of the current paper.

When PAOs think about collaboration with politicians etc., they should critically consider their own role and underline their core values—representing patients and advocating their interests. Emphasizing these values highlights the moral character of PAOs’ work and the moral character, in turn, creates the basis for the claim that PAOs should not only consider their direct target group but also others in the domain of health. The PAOs are encouraged to go beyond their own interests and to see themselves in a broader social context. Understood in this way, the addressees of PAOs’ responsibility covers collective and prospective dimensions.

### The normative standard

If responsibility is assigned to PAOs, a normative judgement is rendered on their activities in relation to a normative standard [35]. Typical standards for attributing responsibility are, for example, legal frameworks or ethical principles. Which standard is chosen depends, inter alia, on the concrete situation in which the subject is located, the activities being judged and the type of responsibility (e.g. legal, political or moral) being considered. If PAOs are seen as morally responsible for patient representation and advocacy, the question remains on which standards this can be claimed.

The PAOs’ demand for more patient participation in research and health policy has been increasingly recognized both legally and politically in recent decades, particularly in Europe [55, 56, 58–60]. Governments are committed, for example by the WHO, to establishing structures that enable the involvement of groups such as disease-specific advocacy organizations [56]. The way in which PAOs are supported varies greatly from country to country and the legislation is often not properly enforced [9]. However, despite this inconsistent legislative landscape, there is a tendency to see PAOs as responsible for representing the interests of the patients. Institutions, such as ethics councils, also give statements about patient and public participation in healthcare. The British Nuffield Council on Bioethics [61], the French National Consultative Ethics Committee on Health and Life Sciences [62] and the German Ethics Council [63] are examples of these and support patient and public participation as they regularly consult affected groups [64]. Insofar as laws, policies and institutional statements assign PAOs certain tasks and enable them to implement patient participation, they can serve as a normative basis for attributing responsibility for patient representation and advocacy to PAOs.

However, although social and political institutions attribute the responsibility for patient representation and advocacy to PAOs, the assignment of this responsibility comes primarily from the PAOs themselves, because the PAOs have assigned themselves this role. Looking at the PAOs’ own statements and constitutions can, therefore, help to identify the normative principles for attributing this responsibility. The constitutions of the PAOs usually define their tasks, missions and core values. Consequently, it would be helpful to examine what role each PAO assigns to itself and which specific responsibilities are associated with this. A PAO that promotes patient advocacy on political committees, for example, has different responsibilities than one that supports patient involvement in clinical trials. Nevertheless, if the common goals and core values behind these specific aims are considered, normative principles can be identified.

The common mission of PAOs to campaign for those affected can often be traced back to the experience of injustice, as many PAOs represent, for example, groups that are stigmatized or diseases that are not sufficiently recognized [1, 3]. One core value that can be identified in the PAOs’ statutes is, consequently, social justice. Furthermore, the wish to help each other and the benefits for their own group as well as for others might be strong motivations for PAO members to join their organization. Mutual support and empowerment are values that are strongly represented by the PAOs. By considering the common goals and core values of the PAOs, the principles of justice, beneficence and empowerment emerge. These bioethical principles can capture the PAOs’ motivations, form the normative basis for their role and work and therefore for their responsibility. While these principles provide a general ethical orientation, they also leave considerable room for interpretation. Although the principles need to be concretized and weighed against each other in specific situations, PAOs can be encouraged to emphasize these ethical principles in their work and consider the implications of their activities regarding these principles.

If the PAOs are assigned responsibility, a normative standard is needed: Legal and political frameworks, but also the PAOs’ own constitutions and the ethical principles of justice, beneficence and empowerment contained therein can be used. Which standards are used may vary depending on the circumstances, in which the PAOs find themselves. The collective dimension can be seen in standards that are the result of a shared deliberative process. The constitutions of PAOs might be assumed to have been elaborated and developed in such a joint process. At least, the ethical principles behind allow room for such processes. If the normative standards also show a long-term orientation, as it is often the case with PAO statements, there is additionally a prospective dimension.

## Responsibility as a tool to structure situations

**Fig. 1:**
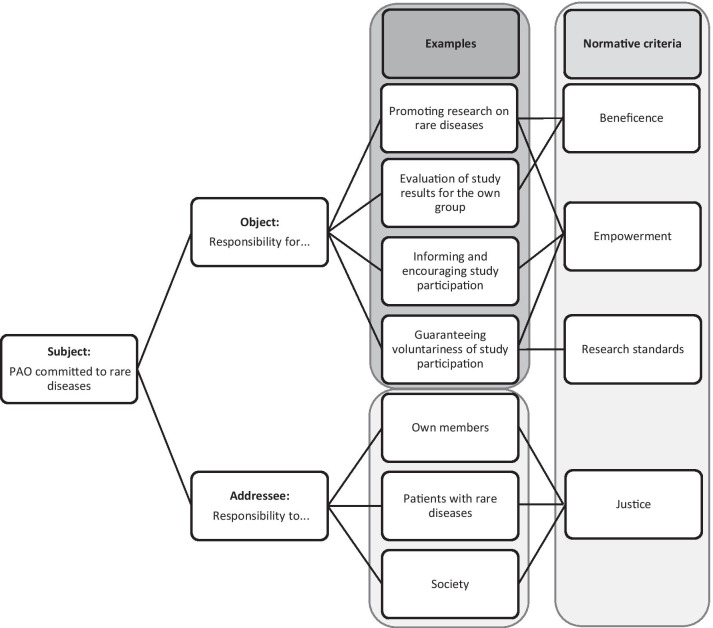
PAOs’ responsibility regarding research

The PAOs can play an important role in the planning and conducting of biomedical research. Many organizations have added contribution to research on their agenda and patients participation, for example, in the design of a research project is usually considered as ethically important in the current bioethical literature [4]. However, PAOs that want to conduce to research find themselves in difficult decision-making situations and are confronted with questions of responsibility. The following example—constructed on debates in the literature and team discussions—demonstrates how the proposed framework of responsibility can serve as a practical tool to structure morally difficult situations (Fig. 1).

A PAO that is committed to rare diseases on a national level receives the invitation to join a clinical trial carried out by a public research institution together with a pharmaceutical company. The PAO could support the study by informing and inviting its members to participate. However, the PAO’s officials are unsure whether they should recruit participants for the study. They are questioning for what and to whom the PAO is responsible in such a situation, and which normative principle can justify this responsibility. The outlined framework can help to structure the situation.

*Regarding the object*, the PAO can emphasize its role: representing persons affected by rare diseases and advocating their interests. These interests consist, at least in the context of research, in promoting studies on rare diseases that result in findings, which helps people regarding diagnosis, therapy or coping with their diseases. It would therefore be the responsibility of the PAO to assess whether the support of this study meets these shared interests. The underlying norm of this responsibility is *beneficence*: the research to be supported is meant to help those affected. If the PAO does not observe the ethical principle of beneficence when selecting the research it wants to endorse and, for example, promotes a study that is not for the benefit of rare disease patients, the PAO may lose the trust of its members and its decision-making power. The principle of *empowerment* complements this obligation, since it is also the responsibility of the PAO to support and empower those affected; which can mean to encourage them to take a (more) active role in research processes. In advertising the study, the PAO would meet this responsibility by informing its members about current research, bringing those affected and scientists closer together and embolden its members to take a position on this research.

When assessing the study, the PAO can also consider *the question of the addressee*: Will the study only serve the group represented by the PAO or will the study have additional collective benefits, for example, for future patients, other social groups or the society? It would be the responsibility of the PAO to include not only its own group but also other addressees in the assessment. The ethical principle behind this responsibility is *justice*. According to this norm, the PAO should consider how access to and benefits of the research are distributed. In line with the PAO’s mission, projects that facilitate the development and improve equitable access and distribution of rare disease treatments should be promoted. However, the PAO may consider whether it is worth investing in this individual research project or whether it would be more effective to support the development of research infrastructures in the field of rare diseases in general.

If the PAO decides to forward the invitation to participate in the study to its members, it would be a further responsibility of the PAO to ensure that the members do not feel any pressure to answer this invitation. The underlying ethical principle is *empowerment* or in a broader perspective respect for autonomy. The offer to participate in the study would probably be better accepted by the members if it was offered by the PAO and not by the pharmaceutical company. However, the PAO is responsible for ensuring that the voluntariness of the invitation is guaranteed and that the participants are sufficiently informed about the context of the invitation, for example, about the relationship between the PAO and the research project partners. In addition, the PAO’s responsibility to its members can be justified by the Declaration of Helsinki [65], which emphasizes, among other research standards, the voluntariness of research participation.

The aim of this case is to illustrate the application of the four-sided model of responsibility. As the application has shown, the interpretation of responsibility regarding the PAOs’ involvement in research is multifaceted and the relata of the model are often interwoven. These ambiguities can be minimised by a precise specification about who is responsible, for what, to whom and on the basis of which ethical standard. An accurate application of the model can help structuring the situation, clarifying the underlying ethical principles and thus contributing to the solution of the conflict. The four-sided model of responsibility, including collective and prospective dimensions, does not claim to be sufficient for all applications, but it can help in structuring and giving orientation.

## Conclusions

This contribution provides an analysis of PAOs’ moral responsibility. Focusing on the *moral* responsibility directs the attention to the moral character of PAOs’ work. PAOs are more than just lobby groups: They are structured in such a way that they are moral agents—hence they are accountable for their actions and have to consider the implications of their activities. The PAOs’ task is relatively clear: To represent those affected and stand up for their rights. This can hardly be taken over by an individual but requires collective efforts. PAOs are voluntary groups in society that have accepted the delegation of responsibility for the presentation of patients, therefore, they are answerable to their target groups but also toward others and the society for the successful execution of this and any deficiencies.

By encouraging PAOs to emphasize their core values, the current analysis can help PAOs to find their own position in difficult decision-making situations. The relational responsibility model is a practical analytical tool that can help PAOs to structure situations characterized by question of responsibility and identify the underlying values. Therefore, it can give PAOs general ethical orientation, help them to find their own attitude and establish clear relationships, for example, with industrial or political agents. Correspondingly, the application of the model can help policy makers, biomedical researchers, and economic stakeholder to understand the roles and responsibilities of PAOs more clearly, which in turn, can help to develop fruitful working relationships with PAOs.

## Data Availability

Not applicable.

## Abbreviations

CSO: Civil society organization
PAO: Patient advocacy organization
WHO: World Health Organization
